# The effects of releasing early results from ongoing clinical trials

**DOI:** 10.1038/s41467-021-21116-4

**Published:** 2021-02-05

**Authors:** Steffen Ventz, Sergio Bacallado, Rifaquat Rahman, Sara Tolaney, Jonathan D. Schoenfeld, Brian M. Alexander, Lorenzo Trippa

**Affiliations:** 1 Dana-Farber Cancer Institute, Harvard T.H. Chan School of Public Health, Boston, MA USA; 2 University of Cambridge, Cambridge, UK; 3 Dana-Farber Cancer Institute, Harvard Medical School, Boston, MA USA

**Keywords:** Cancer, Clinical trials, Randomized controlled trials

## Abstract

Most trials do not release interim summaries on efficacy and toxicity of the experimental treatments being tested, with this information only released to the public after the trial has ended. While early release of clinical trial data to physicians and patients can inform enrollment decision making, it may also affect key operating characteristics of the trial, statistical validity and trial duration. We investigate the public release of early efficacy and toxicity results, during ongoing clinical studies, to better inform patients about their enrollment options. We use simulation models of phase II glioblastoma (GBM) clinical trials in which early efficacy and toxicity estimates are periodically released accordingly to a pre-specified protocol. Patients can use the reported interim efficacy and toxicity information, with the support of physicians, to decide which trial to enroll in. We describe potential effects on various operating characteristics, including the study duration, selection bias and power.

## Abstract (web summary)

Sharing early evidence, as a trial is ongoing, is fundamental for both physicians and patients to make enrollment decisions. Here, the authors report the results of a simulation study evaluating the potential effects of early release of interim efficacy and safety data on the duration and validity of an ongoing clinical trial and demonstrate that positive interim results may shorten trial duration through increased enrollment.

## Introduction

Randomized clinical trials (RCTs) are the gold standard for evidence-based medicine. Unfortunately, however, many RCTs in oncology suffer from slow accrual, in part because many potentially eligible subjects decline to participate^1–3^. Although the motivations for participating in clinical trials are complex and multifaceted, patients who enroll in trials commonly cite their hope for helping others and for receiving a better treatment^4,5^. Here we consider trial policies that could help patients in their enrollment decisions by sharing early treatment-response summaries, such as treatment effect estimates and toxicity-side effect measures, during ongoing clinical studies.

There are ethical arguments for informing clinical trial participants about research findings^6^. It has been documented that patients value the dissemination of final results, and many physicians strive to communicate results in a way that is not sensitive to potential misinterpretation^7,8^. Creating guidelines for how and when to communicate study results is a key problem that several groups have considered. One example is the multi-stakeholder Children’s Oncology Group Returns of Results Task Force^9^, which revealed a significant tension between the need for speedy and rigorous vetting. Most of this discussion, however, focuses on reporting results months after trial completion. But what about sharing early evidence, as the trial is ongoing, which could help patients in their enrollment decisions?

It is standard practice for clinical trials to follow policies, in which evidence accumulated during the trial does not become public before the end of the study. We will call these trials and policies *impermeable*, while we use the term *permeable to* indicate trial designs with a plan to release data summaries at pre-specified time points during the study. The main argument for permeability is that it would allow physicians to share with patients preliminary summaries of efficacy and toxicity endpoints, permitting them to make more informed decisions about enrollment. Patients weigh treatment benefits and risks differently;^10^ for example, side effects such as hair loss may be considered unacceptable by some patients but not by others.

There is a potential benefit of early release of clinical trial data in terms of informed patient decision-making. On the other hand, the use of permeable policies raises issues regarding the feasibility and the scientific integrity of the trial. Permeability does not preclude the use of blinded randomization, but it can affect relevant trial characteristics. For example, the release of unpromising data summaries can reduce the enrollment rate^11,12^, and the study could terminate because of low accrual^12^. Also, the release of preliminary evidence of treatment effects, may lead to asymmetric drop-outs in the control and experimental arms^13,14^. Thus, the main obstacle for increasing the use of permeable studies in clinical research is a concern over the potential for introducing bias in the final analyses.

The public release of limited information during the trial, based on early data, is frequently planned and, in some cases it is a legal requirement^15^. For example, in several trials the data and safety monitoring board decides at periodic interim analyses to continue or terminate the trial, based on early data. The risk that these decisions might be inadequately communicated or misinterpreted by stakeholders has been discussed^15^. Also, the US Federal Regulation CFR 312.56^16^ specifies responsibilities for sponsors of clinical trials, and it requires to notify investigators and the FDA if there are safety concerns^15^.

Reporting early results increases the complexity of data management and trial design. Investigators must specify what information is shared and how often, while maintaining rigorous data vetting standards. There are additional risks of low accrual rates, and potential misinterpretation of the released summaries of efficacy and toxicities^17,18^. On the other hand, the release of interim data summaries could affect clinicians’ recommendations and patients’ decisions and accelerate accrual in the most promising trials, an effect similar to that seen with response-adaptive designs^19–22^. Unlike response-adaptive randomization, however, accrual variations would not be driven by algorithms that assign patients to experimental and control arms but determined by the patients’ reaction to early summaries of clinical trial results.

**Fig. 1:**
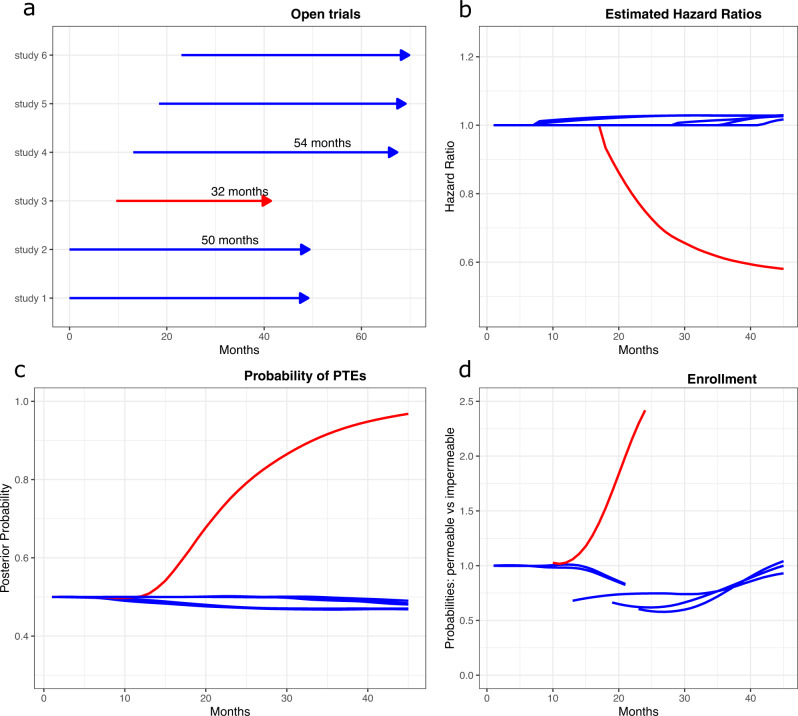
Selected operating characteristics of the permeable environment. A simulation study starts with two open trials, then four additional two-arm controlled trials are opened after 10, 13, 18, and 22 months. The experimental arm in trial 3 (red line) improves median survival time. Experimental arms in the remaining five trials (blue lines) are ineffective with survival distributions identical to the standard of care (SOC). Panel **a** shows the average trial duration across simulations. The remaining panels show for each study the **b** average hazard ratio estimates across simulations, **c** the average posterior probability of a positive treatment effect (PTE) in trial *j*=1,…,6, and **d** the ratio between average enrollment probabilities in permeable and impermeable environments during fixed time windows. The red line in panels **b**–**c** indicates the 3th trial, wich in this simulation study is the only trial testing an effective experimental treatment. Results are based on 10,000 simulations of the six trials.

Here, we perform a simulation study to understand the impact of permeable policies on clinical trial operating characteristics. We considered scenarios in which independent studies update efficacy summaries following a consistent protocol (Fig. 1), and patients may use these reports to decide which trial to enroll in. Similarly, in a multi-arm study each patient may select and restrict randomization, on the basis of early data summaries, to a subset of potential treatment arms. Here, mandating randomization to the control arm of the multi-arm study allows the investigators to estimate treatment effects.

## Results

Figure 1 illustrates some characteristics of a simulated environment with multiple trials that release monthly interim data (permeable environment). Six two-arm studies enrolled patients (panel a) and only one experimental treatment (red arrow) had a positive treatment effect compared to the standard of care. Panels b and c show the averages, across simulations, of trial-specific HR estimates and of the posterior probabilities of positive treatment effects over time. Panel d illustrates, for fixed time windows that follow the opening of each trial, the ratio between the average enrollment rate in the permeable environment and the average rate in an impermeable (no early release of treatment summaries) environment.

Table 1 provides a summary of assumptions for our simulations of glioblastoma (GBM) studies in the setting of early release of data summaries (permeable environment) versus the current standard (impermeable environment). Each simulation generated 30 clinical studies with open enrollment at different time points during a 10-year period. The main difference in the simulation of trials in the permeable and impermeable environments was that early data summaries were disclosed only in the former.

**Table 1:** Assumptions used in the simulation study to model permeable and impermeable clinical research environments in GBM.

Parameter	Description
Simulation period	120 months
Enrollments	On average a total of 53 enrollments per month to the open trials.
	6 trials open at time 0, the remaining 24 open at random time points during a 120 months period.
Effective drugs	3 of the trials test an effective experimental treatment
	(3 studies, selected randomly among studies 6,7,…, 29).
Median survival	10 months for the standard of care and each ineffective drug,
	14.3 months for effective treatments.
Sample size	224 patients per study.
Follow-up	Until 144 events have been observed.
	Approximately 80% power to detect a hazard ratio of 0.7 at *α* = 0.1
	Permeable Environment
Release of information	Monthly release of estimated hazard ratios and posterior probability of positive treatment effects.
Enrollment	Summary statistics modify the study-specific enrollment rates.
	Impermeable Environment
Release of information	No summary statistics are released before completion of the study.
Enrollment	Open trials have equal enrollment probabilities.

### Bias, type I error, and power

As expected, under the outlined assumptions, permeable and impermeable environments generated nearly unbiased treatment effects estimates and had similar probabilities of positive results (approximately 80% power) with identical type I error rates (see Supplementary Table S2).

### Time to complete clinical trials

**Fig. 2:**
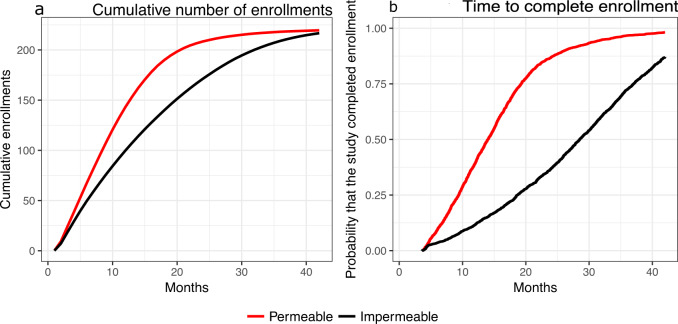
Enrollment time in permeable and impermeable environments. The average cumulative number of enrollments (**a**) in permeable and impermeable environments and the distribution of the time to complete enrollment (**b**) for the first study that evaluates an effective treatment.

The average time to complete trials testing effective or ineffective treatments differed in permeable and impermeable environments. Figure 2 illustrates the trial-specific enrollment rate and the time necessary to test the experimental treatment. In our simulations, the first trial that evaluated an effective treatment in the permeable environment completed enrollment on average 15.6 months after it started enrolling patients. By comparison, the impermeable environment required on average 28.0 additional months, a substantial increase in the trial duration. Symmetrically, we observed that permeable trials with ineffective experimental treatments required, on average, three additional months for completion compared to impermeable studies.

When we reduce the frequency of the release of data summaries from 1 months to 2, 3, 6, 9 or 12 months, the average enrollment period of trials with effective experimental arms increases from 15.7 months (monthly release) to 19.6 months (release every 12 months), compared to 28 months for impermeable trials, respectively (see Supplementary Table S2). The type I error rates and power are not affected by the frequency of the release of data summaries.

The duration of permeable trials is strictly connected with the behavioral model used to link the release of data summaries and the subsequent variations of the enrollment rate. We observed analogous differences between the duration of permeable and impermeable studies, as those that we described, when we considered different parametrizations of the model. But the magnitude of these differences changes across parametrizations (Supplementary Figure S2, panel b).

Similar results were obtained in simulations of multi-arm platform designs (see the Supplementary Material).

### Sensitivity analysis

We considered several deviations from the idealized model, perturbing the patient decision-making model to enroll into studies, considering patient subgroups that would react differently to early estimates of efficacy and toxicity, and illustrating potential pernicious incentives of various stakeholders in a permeable environment.

We first generated permeable trials in which some of the experimental treatments could be obtained off-label. A considerable proportion of patients would likely obtain off-label treatments instead of enrolling in a randomized study when experimental trials disclosed promising early results. This scenario (Supplementary Fig. S1, Panel a) indicated sensitivity of the time to complete enrollment and the risk of reducing the overall accrual rate in the open trials.

We then considered the impact of early release of misleading information that is inconsistent with the data generated in the ongoing trials. This scenario reflects a possible incentive of stakeholders, such as trial sponsors, to inflate the reported probability of a positive effect to secure a higher enrollment rate. In our simulations, we assumed that in one study testing an ineffective treatment the investigators misreport the evidence of a treatment benefit. The results (Supplementary Fig. S1, Panel b) point to marked sensitivity of study-specific accrual rates to misreported summaries.

We also found that permeability policies can lead to differences in the populations of subjects enrolled across trials. In a simulation, we considered two groups of patients with different prognostic profiles. For patients in Group 1, trial selection was influenced by early data summaries, while patients in Group 2 selected their trial at random. Under this scenario, we observed noticeable trends (Supplementary Fig. S1, Panel c) in the proportions of enrolled patients in each group, both in trials with effective and ineffective treatments. Patient subpopulations could be significantly overrepresented or underrepresented in permeable trials. Variations in the composition of the enrolled patients over time could lead to biased treatment effect estimates for the overall population when the effects vary across subgroups.

Furthermore, in the permeable environment, we examined the possibility that participants might dropout of a trial if discouraging information is released while the trial is ongoing. We considered again two groups (Group 1 and Group 2) with good and poor prognoses, respectively, and incorporated distinct dropout propensities for patients in these two groups. We considered two scenarios where Group 1 or Group 2 had a higher dropout propensity. We observed bias, for example, in the estimated median survival (Supplementary Fig. S1, Panel d). When patients in Group 1 had higher dropout propensity, standard Kaplan–Meier estimates tended to underestimate the median survival and vice versa.

Finally, we considered the risk of trial discontinuation and the resulting decrease in power due to early release of negative results. We included in our model of individual enrollment decisions (Supplementary Material) a threshold on the reported probability of positive treatment effects, below which patients would refuse to join the trial. If this threshold is high, an effective trial with poor initial results might not enroll patients and close early because of low accrual. In our simulations, this effect decreased the power of the trial (Supplementary Fig. S2, Panel a). A pragmatic strategy to mitigate this risk would be to wait and release efficacy/toxicity estimates once they match a desired level of accuracy, for example, a confidence interval with a pre-specified length.

## Discussion

Periodic analyses, similar to those already in use in response-adaptive trials, can generate real-time data summaries that can be shared with physicians and/or patients to inform enrollment decisions. Here, we evaluated the effects of early release of clinical trial data as an alternative to the standard impermeable practice of not disclosing interim efficacy and toxicity data from ongoing clinical trials.

Informing patients participating in clinical trials about early efficacy and toxicity data could have significant benefits. Importantly, increased permeability can accelerate enrollment of trials evaluating effective treatments.

However, sharing preliminary results is also associated with risks of compromising the statistical validity of the study conclusions. There are areas of concern including the effect of misreporting early results and uncertainty on how patients react to early data summaries. Our simulation study identified risks associated with permeability. The release of negative data summaries (e.g., a negative estimate of the treatment effects) in a permeable study can reduce the enrollment rate. Moreover, the study could terminate because of an insufficient number of enrollments^12^. Also, in unblinded RCTs, the release of early evidence of treatment effects, may increase the number of drop-out decisions in the control arm, particularly if the experimental treatment is available off-label^13,14^.

Different groups of patients may react differently to interim results. This could alter the demographics and clinical profiles of enrolled patients during the trial, and compromise the generalizability of study results. It is also worth noting that the populations enrolled in distinct trials may be different both in permeable and impermeable environments.

The release of interim data summaries during an industry-sponsored trial may also have economic implications for pharmaceutical companies. Pharmaceutical companies might have more incentives to release promising information then discouraging early data. Nonetheless, a transparent plan should be specified before the onset of the study when and which early summaries information will be released.

Operational bias arises when the trial conduct or subjective decisions during the study affect the validity of the statistical conclusions^23,24^. The release of data summaries can impact on relevant post-enrollment decisions (e.g., drop-out decisions), potentially contributing to bias the study results. This risk correlates with the duration of the treatment, the period between randomization and the measurement of the primary outcome, and other aspects that are specific of the disease setting. We provide two examples.

*Example-1*. An open-label RCT in head and neck cancer, where the treatment is administered during a period of several months, and the primary outcome (overall survival) has a median of >5 years. The release of early summaries may induce asymmetric drop-out decisions in the experimental and control arms^13,14^. Therefore, in this example permeability might bias the study results.

*Example-2*. A blinded RCT in intensive care units that test an experimental treatment to prevent infections. The treatment is administered in a short period of a few hours, and the outcome is available within days from randomization. In this example the risk of bias is considerably lower. Moreover, data summaries can be release at conveniently planned times, avoiding overlap with the short period between patient randomization and the measurement of the primary outcome.

Any potential advantage of a permeable environment would also be sensitive to the quality of reporting and interim analyses. Early estimates should be comparable across experimental treatments and easy to interpret. In our simulations of permeable clinical trial environments, we observed potential benefits in terms of speed in the development of new treatments. A permeable environment could be centered on multi-arm platform trials where patients can restrict their randomization to individually selected subsets of arms. Another possibility is a system where regulators provide a mechanism, similar to Clinicaltrials.gov, to communicate data summaries from ongoing trials.

Several designs can be considered for permeable trials. For example, the choice of the data summaries and the time to release information can differ across candidate designs. The operating characteristics, potential benefits and risks of candidate designs can be described using simulation models and sensitivity analyses. The characteristics of the trial design should be discussed among stakeholders, including investigators, patient-advocates and sponsors, and should be compared with impermeable designs. The choice of a permeable design has to combine the control of risks that may arise with permeability, including bias, and the release of up-to-date information during the study.

In consideration of the lack of experience with permeable study designs, their initial applications should be gradual, starting from study designs, treatments and trials that present low risks associated with permeability. For example, initial applications could plan the release of selected data summaries at a mature stage of the trial. This would permit the release of summaries with limited uncertainty, which are likely to be aligned with the final results of the trial. Also, initial applications of permeable study designs could target trials in the early stages of the drug development process, and in disease settings where primary outcomes become quickly available after randomization. Moreover, applications should be directed to double blinded studies. Our sensitivity analyses suggest these cautious criteria for initial applications of permeable trial designs. The primary motivation is the need of preserving the scientific validity of the drug development process. These cautious criteria mirror risks that emerged in sensitivity analyses, including drop-out decisions, and the risk of a rapid reduction of the enrollment rate during the study.

Initial experiences with permeable designs can generate data on how patients and other stakeholders react to the release of early data summaries during clinical trials. This includes possible variations of the enrollment rate after the release of promising or discouraging summaries. Datasets to link, in specific disease settings, the release of summaries during the trial and the subsequent enrollment or drop-out decisions, would be relevant to optimize permeable trial designs. In particular, they can support decisions on when and which data summaries can be released.

The publication of RCTs results in medical journals is a key component of clinical research^25^. The peer-review process^26,27^ scrutinizes the trial design, statistical analyses, and the reported results. One of the aims of this process is the publication only of studies with limited risks of bias and an accurate control of false positive findings. This further suggests the application of permeable designs only in trials and disease settings where permeability presents low risks and minimal concerns of jeopardizing the scientific validity of the study. Furthermore, initial applications of permeable designs can generate discussions and data to refine the release of data summaries in future studies.

Permeable designs release data summaries to inform patients and physicians. This goal has connections with previous work that investigated relevant factors that impact on patient decisions to enroll or decline to enroll into clinical studies^3–5,28–33^. These includes patient’s expectations of clinical benefits^5,28,29^, which in some cases are highly optimistic^4^. Patients that decline to enroll into trials often express concerns associated with limited knowledge of the experimental treatment, about potential toxicities^32^, impact on their quality of life^31^ and about randomization^4,31^. Drop-out decisions are often the result of side effects^32^ or disease progression^32^. Several studies emphasized the importance of making accessible to patients interpretable information on the experimental treatment^3–5^.

In our simulation study, in absence of previous applications of permeable designs, we explored plausible relations between the release of data summaries and enrollment decisions. We specified a behavioral model, to mimic potential variations of the enrollment and drop-out rates during the trial. Our study considered a range of parametrizations of the model, and a few relevant differences between permeable and impermeable studies were consistently present across parametrizations. For example, when the experimental treatment has positive effects, permeable designs tend to reduce the trial duration.

The simulation study revealed the effects of early release of clinical trial data on key operating characteristics. New designs of clinical studies with release of early data summaries need to consider how and when to release information, and the organizational, ethical, and statistical implications of doing so. Implementation of permeable policies to inform physician and patient enrollment decision making will require a careful consideration of the appropriate communication of trial data, along with potential bioethical considerations of a permeable environment and the impact on trial accrual and integrity. Solving these issues could enable investigators to better account for individual preferences of patients in clinical research.

## Methods

### Simulation models

We specified two models (see Supplementary Material for details) of clinical research. In model 1, independent two-arm controlled trials followed a detailed protocol to disclose early estimates during the study. Model 2 was a platform trial^34,35^ with several experimental arms and periodic release of information on early efficacy/toxicity estimates for each of the experimental treatments. In this platform study model, patients at enrollment were allowed to choose from a catalog of treatments. Each patient could restrict randomization to the control arm and any subset of experimental arms. The randomization probabilities to the control and to each experimental arm selected by the patient were identical.

We first conducted simulations using stylized assumptions ideal for permeable trial policies. We then performed sensitivity analyses, considering several scenarios where permeability could have a deleterious effect on specific trial operating characteristics and the final statistical analyses of the trial data.

### Simulation parameters

We tailored simulations to a specific setting, phase II studies in GBM, using realistic simulation parameters (see Table 1) from a systematic review of the literature^36^, including primary outcome (overall survival), median survival times, and enrollment rates. The two-arm trials in the simulations were assumed to have balanced randomized designs with identical sample sizes for the experimental and control arms.

### Periodic release of summary information

We considered the periodic (monthly) release of various summaries of preliminary data, such as point estimates of the hazard ratio (HR) between the experimental and control arm for each trial, or the posterior probability of a positive treatment effect. Additional and complementary summaries released monthly for each treatment could include probabilistic predictions of response, median survival, or severe adverse events.

### Patient decisions and enrollment probabilities

We modeled individual patient decisions assuming that patients would be more likely to enroll in studies with promising early summaries released during the accrual period. In Model 1, the probability $p_{j,\ell }$ that a patient selects study *j*, during month ℓ, was proportional to an increasing function *g* of the posterior probability of a positive treatment effect (PTE) of the experimental treatment in study *j*, $\pi _{j,\ell } = \Pr \left( {HR_j < 1\left| {Data\,at\,month\,\ell } \right.} \right),$ where *HR*_*j*_ is the HR between the experimental and control arm in study *j*. We compute the posterior probabilities $\pi _{j,\ell }$ for each open trial using a normal prior $(mean:0,variance:\,0.5)$ for log(*HR*_*j*_). The Supplementary Material provides additional details on the definition of *g*. We used a probability model ($p_{j,\ell }$) for the patient’s enrollment decisions, as physicians’ recommendations and enrollment decisions are likely to depend on additional factors, such as the number of visits required or proximity to the closest trial site. In Model 2, the patient was allowed to select from a list of treatments and randomization was restricted to this list. The early data summaries would influence the probability that the patient selects an available treatment *j*. Similar to Model 1, we modeled the probability of selecting treatment *j*, during month ℓ, as an increasing function of the PTE of experimental arm *j* compared to the control arm (see Supplementary Material for details).

### Clinical trials without periodic release of early estimates

For comparison, in the impermeable environment, in our simulations, the probability that a patient would enroll in a trial was identical across all open trials. Similarly, in a platform study that allowed individual selection of experimental arms at enrollment, as in Model 2, but without periodic release of early data summaries, our simulations fixed the selection probabilities, which were identical across arms.

### Estimation and testing of treatment effects

Point estimates and testing of treatment effects at completion of simulated clinical trials were based on the Cox proportional hazards model^37^ and the log-rank test^38^.

### Sensitivity analyses

In initial simulations, we assumed that the number of patients that enroll each month to one of the open trials (Model 1) would be approximately constant. Similarly, the overall rate of enrollment in the platform study (Model 2) would not vary during time. In sensitivity analyses, we relaxed this assumption and also considered misreporting of early summaries, as well as potential patient dropout from studies associated with the early release of data summaries. Additional sensitivity analyses are described in the Supplementary Material.

Permeable designs have not been previously used. We specified a behavioral model to link the release of data summaries during the trial and the subsequent enrollment decisions. Our simulation study includes a range of plausible parametrizations of the model. We report differences between permeable and impermeable studies that were consistently present across parametrizations. As expected, relevant operating characteristics, including the trial duration, are strictly connected and sensitive to the parameters of the behavioral model (the Supplementary Material includes several parametrizations). For this reason, the comparison of permeable and impermeable designs required the exploration of different parametrizations of the behavioral model the related the released data summaries with patients’ enrollments decisions (see the Supplementary Material).

### Reporting summary

Further information on research design is available in the Nature Research Reporting Summary linked to this article.

## Supplementary information

Supplementary Information

Reporting Summary

Supplementary Code

## Data Availability

Simulated datasets were generated in R, version 3.3.0. The R code provided in the Supplementary file Supplemantary Code can be used to generate the data used for the analysis.

## Code availability

R code to replicate the analyses is available in the Supplementary file “Supplemantary-Code.zip”.
