# Supplementary material for: The effects of releasing early results from ongoing clinical trials
Source: Nat Commun. 2021 Feb 5;12:801. doi: 10.1038/s41467-021-21116-4 (PMC7864990; doi:10.1038/s41467-021-21116-4)
Supplement: Supplementary file 1 — Supplementary Information [file 41467_2021_21116_MOESM1_ESM.pdf]

SUPPLEMENTARY MATERIAL
“The Effects of Releasing Early Results from Ongoing Clinical Trials”

Steffen Ventz[1]*, Sergio Bacallado[2], Rifaquat Rahman[3], Sara Tolaney[3], Jonathan Schoenfeld[3], Brian Alexander[3] and Lorenzo Trippa[1]

[1]Dana-Farber Cancer Institute, Harvard T.H. Chan School of Public Health, Boston, MA, USA,

[2]University of Cambridge, Cambridge, UK

[3]Dana-Farber Cancer Institute, Harvard Medical School, Boston, MA, USA

December 8, 2020

To investigate the implications of permeability, we consider two models for clinical research. The first, discussed in Section S1, is one in which independent controlled clinical trials follow a detailed protocol to disclose periodically data summaries. The second model, in Section S2, is a platform study with several experimental arms, in which the organization conducting the study periodically releases information, again following strict protocols. In this second setting, patients are allowed to choose from a catalog of randomization distributions: patients could, for example, accept the possibility of being randomized to the control and some of the experimental arms, while denying consent to receive other experimental treatments based on interim summaries.

We conduct simulations, using stylized assumptions, to compare permeable and impermeable research environments. The simulation study is tailored to a specific setting, phase II studies in Glioblastoma, with the goal of evaluating the potential consequences of more permeable designs, under realistic simulation parameters derived from a systematic review [5], (Table 1).

*steffen@jimmy.harvard.edu

S1 Independent trials releasing efficacy information

We provide a detailed description of the models for permeable and impermeable research environments in which independent trials enroll participants from the same pool of patients. We shall consider fixed a time period of T months and focus on a single disease. Alternatively the time horizon could terminate with the discovery of the first effective treatment. The trials are assumed to have identical balanced randomized two-arm designs with overall survival endpoint and a planned enrollment of n patients. At time $t = 0$, a total of $m \geq 1$ clinical trials evaluate treatments. These trials may be either enrolling patients or in the follow-up stage. At random times, $0 < t_{m+1} < t_{m+2}, \ldots$, new studies will open. The rate λ_P models the average number of enrollment per month to active trials. Efficacy is tested using a log-rank test [2] with null hypotheses $H_j : HR_j \geq 1$, where HR_j is the hazard ratio between the experimental arm and the standard of care (SOC) in the j-th trial.

Permeable Environment

In the permeable environment, each open trial releases early data summaries at the end of every month $\ell = 1, 2, \ldots, T$. But the primary hypothesis H_j of each trial j can only be rejected at completion of the trial. For this reason, testing H_j (one of the primary purposes of the trial) does not involve adjustments for multiplicity (e.g. α spending-functions) to control the type I error rate. We may consider communicating various summaries of preliminary data, for example, point estimates of the hazard ratio $\widehat{HR}_{j,\ell}$, or the posterior probability of a positive treatment effect (PTE) $\pi_{j,\ell} = \Pr(HR_j < 1|$ Data at month $\ell)$. The first statistic conveys the magnitude of the effect, while the latter quantifies the degree of uncertainty on a positive effect. In our simulations, we compute the posterior probabilities $\pi_{j,\ell}$ for each open trial with a normal prior on $\log(HR_j)$ having mean zero and variance $1/2$. Complementary summaries could include the number of previously randomized patients and predictions of response or survival for each treatment.

It is difficult to evaluate which summaries are most interpretable or to predict how patients and physicians would respond to this information. We define a stochastic model for the patients' enrollment decisions, and evaluate the sensitivity of the simulation results with respect to the parameters of the decision model. In our simulations, we assume that if $\pi_{j,\ell}$ is below a threshold $\theta_L \in [0, 1]$, then patients do not enroll in trial j. Symmetrically, trials with $\pi_{j,\ell}$ above a threshold $\theta_U \in [\theta_L, 1]$ are selected with identical probabilities. The probability that

a patient selects study j, during the period $[\ell, \ell+1)$, is $p_{j,\ell} \propto g_\theta(\pi_{j,\ell}),$ where $g_\theta(\cdot)$ is monotone,

$$g_\theta(\pi) = \begin{cases} 0 & \text{if } \pi < \theta_L, \\ \left(\frac{\pi-\theta_L}{\theta_U-\theta_L}\right)^{\theta_S} & \text{if } \pi \in [\theta_L, \theta_U], \\ 1 & \text{if } \pi > \theta_U, \end{cases} \tag{S1}$$

and $\theta_S \geq 0$. With θ_S large and θ_U close to one patients select the trial with the largest PTE.

Impermeable Environment

In the impermeable environment information on efficacy is released only after the end of the follow-up period of each trial. However, we assume that trials have a degree of adaptivity to early evidence of futility, as in most two-arm trials. Trials with posterior probability $\pi_{j,\ell}$ below θ_L are stopped for futility. The probability that patient i enrolls in trial j is constant across all open impermeable trials.

To simplify comparisons of permeable and impermeable environments we assume that permeable trials that do not recruit patients for a pre-specified period of time ($\pi_{j,\ell} < \theta_L$) are closed.

Scenarios

The following simulation study is tailored to Phase II trials in Glioblastoma (GBM) over a period of 120 months. The parameters used in these simulations (see Table 1 in the manuscript) were selected from our systematic literature review of clinical trials in GBM during the last 15 years [5].

Each month, on average 53 GBM patients enroll in one of the open trials. Five studies are open at the beginning of the simulation period and 25 additional studies are opened at random time points during a 120 months period. The median survival for the control and non-effective treatments is 10 months compared to 14.3 months ($HR = 0.7$) for effective experimental arms. Each study enrolls up to 224 patients and, following standard protocols for survival analysis [3], final analyses are conducted after 144 events have been observed, approximately 12 months after the last enrollment in the study. With 10% type I error rate, $HR = 0.7$ and analyses after 144 events, each trial has approximately 80% power to detect the treatment effects.

In the permeable environment, the parameters for the patient decision model were set to $(\theta_L, \theta_U, \theta_S) = (0.05, 1, 3)$. To provide some interpretation of this choice, consider Panel (C) in

Figure 1 in the manuscripts. After 20 months, there are 5 trials open; one of them evaluates a treatment showing early evidence of efficacy, while the others test treatments similar to the SOC. The posterior probability of a PTE in the effective trial is (average across simulations) around 0.7, and for the other trials it is close to the prior probability of 0.5. Under our model parameters, a patient would be around 3 times more likely to enroll in the effective trial than in any given ineffective trial.

Figure 2 in the manuscript and Table S1 summarize the results of the simulation study. Permeable and impermeable environments have similar probabilities of a positive result for the first effective treatment (approximately 80% power) with identical type I error rates. In the permeable environment 9% of all simulated trials without effective experimental treatments are stopped early. Permeable trials with an effective experimental arm are stopped early in less than 1% of all simulations. Impermeable trials are stopped early for futility with similar frequencies as in permeable studies.

In our simulation, 3 out of 30 experimental arms are effective, and the first effective treatment is tested in study j_{eff}, which ranges between the 6-th, up to the 27-th. Figure 2 in the manuscript shows the completion time of trial j_{eff}. Panel (A) shows the average cumulative number of enrollments during time, starting from the onset of the study, and Panel (B) shows the distribution of the number of months to complete the enrollment across simulations. The illustrated enrollment period goes from the first randomization until the enrollment is stopped because either enrollment in study j is completed or the trial is stopped for futility.

Table S2 reports selected operating characteristics of permeable trials, with early data summaries released every 1, 2, 3, 6, 9 or 12 months (columns of Table S2). When we reduce the frequency of the release of data summaries, the average enrollment period of trials with effective experimental arms increases, from 15.7 months (monthly release) to 19.6 months (release every 12 months), compared to 28 months for impermeable trials. Also, as expected, type I error rates and power are not affected by the frequency of the release of data summaries. Indeed, the null hypothesis (i.e. absence of positive treatment effects) is tested only at completion of the study.

Study	Enrollment period<br>Mean (SD)	Time to final results<br>Mean (SD)	Sample size<br>Mean (SD)	Power	P_{Stop}
		permeable environment			
ineffective trial j_{prev}	27.2 (20.8)	34.5 (22.0)	210 (33)	0.09	0.09
effective trial j_{eff}	15.6 (09.5)	28.8 (09.6)	222 (07)	0.80	<0.01
ineffective trial j_{next}	25.2 (19.7)	33.5 (21.9)	212 (32)	0.10	0.09
		impermeable environment			
ineffective trial j_{prev}	27.2 (11.3)	31.1 (09.3)	212 (27)	0.09	0.09
effective trial j_{eff}	28.0 (12.1)	35.2 (09.9)	221 (06)	0.80	<0.01
ineffective trial j_{next}	26.4 (12.1)	31.4 (11.1)	214 (26)	0.11	0.09

Table S1: Selected operating characteristics of permeable and impermeable environments in GBM. Results are based on 5,000 simulations of a drug-development period of 10 years during which 30 two-arm trials evaluate new experimental treatments in GBM patients. We use $(\theta_L, \theta_U, \theta_S) = (0.05, 1, 3)$ to define the enrollment probabilities in (S3). Study $j_{\text{eff}} \in \{6, \cdots, 27\}$ corresponds, in each simulation, to the first trial that evaluates an effective experimental treatment. Study j_{prev} denotes the last trial that opens enrollment before study j_{eff} is activated. Study j_{next} corresponds to the first study that evaluates a non effective experimental treatment and opens enrollment after j_{eff}. Studies $j_{\text{prev}}, j_{\text{eff}}$ and j_{next} are random and vary across simulations. P_{Stop} indicates the probability of stopping the trial early.

	Frequency of release of early data summaries (months) in the permeable environment					
	1	2	3	6	9	12
			effective trial j_{eff}			
Mean enrollment period	15.7 (9.7)	16.3 (9.5)	16.5 (8.5)	18.1 (10.2)	18.6 (9.1)	19.6 (9.6)
Mean time to final results	28.8 (8.6)	29.1 (8.5)	29.3 (7.5)	30.5 (11.4)	30.7 (9.4)	31.2 (9.2)
Power	0.80	0.80	0.81	0.80	0.80	0.79
P_{Stop}	0.00	0.00	0.00	0.00	0.00	0.00
Mean accrual Rate	0.36 (0.18)	0.36 (0.18)	0.35 (0.19)	0.34 (0.19)	0.33 (0.18)	0.31 (0.18)
			ineffective trial j_{prev}			
Mean enrollment period	26.6 (19.7)	27.5 (21.0)	28.2 (21.1)	27.9 (20.6)	28.2 (19.9)	29.0 (20.2)
Mean time to final results	33.5 (19.4)	34.9 (22.4)	35.3 (22.5)	34.8 (21.1)	35.7 (23.2)	35.8 (21.2)
Power	0.10	0.11	0.08	0.11	0.11	0.10
P_{Stop}	0.08	0.08	0.07	0.06	0.05	0.03
			ineffective trial j_{next}			
Mean enrollment period	25.9 (19.8)	25.4 (19.5)	25.6 (19.4)	26.9 (20.1)	27.3 (19.7)	26.8 (18.4)
Mean time to final results	34.3 (23.7)	33.4 (21.1)	34.1 (22.7)	35.1 (22.8)	36.0 (24.4)	34.7 (21.5)
Power	0.10	0.10	0.11	0.10	0.12	0.09
P_{Stop}	0.09	0.07	0.07	0.05	0.04	0.04

Table S2: Selected operating characteristics of permeable environments in GBM when early data summaries are released every 1, 2, 3, 6, 9 or 12 months. Results are based on 1,000 simulations of a drug-development period of 10 years during which 30 two-arm trials evaluate new experimental treatments in GBM patients. We use $(\theta_L, \theta_U, \theta_S) = (0.05, 1, 3)$ to define enrollment probabilities in (S3). P_{Stop} indicates the probability of stopping the trial early. Values on parenthesis indicate standard errors. Study $j_{\text{eff}} \in \{6, \cdots, 27\}$ corresponds, in each simulation, to the first trial that evaluates an effective experimental treatment. Study j_{prev} denotes the last trial that opens enrollment before study j_{eff} is activated. Study j_{next} corresponds to the first study that evaluates a non effective experimental treatment and opens enrollment after j_{eff}. Studies $j_{\text{prev}}, j_{\text{eff}}$ and j_{next} are random and vary across simulations.

S1.1 Sensitivity Analyses

The stylized assumptions of the previous section do not represent some aspects of a permeable environment, and it is important to understand how departures from the model affect the operating characteristics.

1) Interim results can influence the overall enrollment rate

Many trials test drugs that are already indicated for other diseases and therefore commercially available. This is common in oncology, where a drug approved for a cancer type may be later approved for other malignancies [4]. In this situation, it could be possible for a patient to obtain the drug off-label, instead of enrolling in a randomized study. It would not be surprising if the proportion of patients opting to receive a treatment off-label increased when evidence of efficacy from a trial becomes is released in a permeable environment.

In our sensitivity analysis, we assume a set of treatments $\mathcal{J}$ can be obtained off-label, and we incorporate patients' reactions into our model, with a time-varying overall enrollment rate $\lambda_P(\ell) = \lambda_P \times (1 - p_{OL} \times I(\theta_{OL} < \max_{j\in\mathcal{J}} \pi_{j,\ell}))$. To be precise, $\lambda_P(\ell)$ is the rate describing the enrollment of patients in all trials in the time period $[\ell, \ell+1)$. Here, λ_P is the constant rate used in our basic model, $p_{OL} \in [0,1]$ represents a proportion of patients that obtain off-label treatments when experimental treatments under study show promising results, and $\theta_{OL} \in [0,1]$ is a threshold for the posterior probability of a positive effect above which these patients opt for the off-label treatment.

Panel (A) in Figure S1 illustrates the sensitivity of the time to complete enrollment with respect to this perturbation. It shows the mean time for three studies (j_{prev}, j_{eff}, and j_{next}) to complete enrollment (y-axis) when a proportion of patients p_{OL} (x-axis) in $[0, 0.6]$ prefers to obtain drugs off-label instead of enrolling into active trials if the posterior probability of a positive effect, for any of the treatments j, becomes large ($\pi_{j,\ell} > 0.9$). The mean time to complete enrollment for study j_{eff} increases substantially, from 15.6 months, to 22.5, 32.6 and 57.0 months when $p_{OL} = 15\%, 30\%$ and 50%. For studies testing ineffective treatments the average length of enrollment increases similarly.

2) Misreported interim results

We consider the impact of misleading information which is inconsistent with the data generated in ongoing trials. This reflects possible incentives of stakeholders to misreport the probability of a positive treatment effect in order to secure a higher enrollment rate. We assume that in

one study ($j = 6$) which tests an ineffective treatment, the investigators misreport $\pi_{j,\ell}$ and publish the value $\min(1, \pi_{j,\ell} + \delta)$ with constant misreporting $\delta \geq 0$ during the trial.

Panel (B) in Figure S1 shows the average cumulative number of enrollments in the study with misreporting over time. We compare the model without misreporting ($\delta = 0$) to two different levels of misreporting, $\delta = 0.15$ and 0.3. Even with moderate misreporting ($\delta = 0.15$), the number of patients enrolled in study 6 (testing an ineffective treatment) increases substantially.

3) Interim results determine population trends

We consider a scenario where latent variables define subpopulations. Patients in different subpopulations have different prognostic profiles, and they also react differently to interim results. This could induce distinct population trends in each trial and compromise the validity or generalizability of the trial results. For instance, patients with higher educational levels might be more reactive to interim results; which would lead to trials with early promising data to be enriched with this group of patients. If the educational level is also associated with prognosis or with treatments' effects, then the effect sizes across trials may not be comparable. It is worth noting that the populations enrolling in distinct trials may also be different in an impermeable setting.

We assume that there are two groups of patients, *group 1* and *group 2*, with good and poor prognostic profiles respectively. Experimental treatments can have identical or distinct effects in these two groups. Additionally, patients in *group 1* select trials accordingly to expression (S3), while patients in *group 2* select their trial randomly with identical probabilities across all open studies. Let p_1 be the proportion of patients in *group 1*, and let (μ_1, μ_2) be the mean survival time under standard of care in groups 1 and 2. We consider three simulation scenarios, with $p_1 = 0.2, 0.4$ or 0.6, and $\mu_1 = 40.1, 29.4$ or 18.7. In each scenario we set $\mu_2 = 8$. In our simulations, superior treatments have identical effects (within stratum $HR = 0.7$) in groups 1 and 2.

Panel (C) in Figure S1 shows the proportion, across simulations, of patients in groups 1 and 2 during the enrollment period of the trial j_{eff}, from the first enrollments until the last ($n = 240$) one. The sensitivity analysis shows that patient subpopulations could be significantly overrepresented or underrepresented in permeable trials. Variations in the composition of the enrolled patients during time can lead to biased treatment effect estimates when the effects are different across groups.

4) Dropouts due to interim results

In the permeable environment participants in study j might leave the trial when $\pi_{j,\ell}$ becomes small. We consider again two groups (*group 1* and *group 2*) with good and poor prognoses and incorporate distinct drop-out propensities for patients in these two groups. Patients that enrolled into trial j have positive probability to leave the trial when the posterior probability $\pi_{j,\ell}$ becomes smaller than a fixed threshold. The propensity for drop-out can differ across groups $g = 1, 2$.

Panel (D) of Figure S1 shows, for study j_{prev}, bias in the estimated median survival. The median survival time was estimated using Kaplan-Meier estimates, assuming non informative censoring and including drop-out decisions as censoring events. We assumed identical prevalences ($p_1 = 0.5$) of patients with good and poor prognoses (*group 1* and *2*), and median survival times of 17 and 3 months in these two groups for the SOC and ineffective treatments. We consider two cases. In the first one (brown curve), if $\pi_{j,\ell}$ become smaller than the threshold, then each patient in *group 1* drops out with probability equal to $0.05, 0.1, \cdots, 0.9$ (x-axis) and patients in *group 2* drop-out with probability 0.05. Symmetrically, in the second case (black curve) the drop-out probability equals 0.05 for patients in *group 1* and $0.05, 0.1, \cdots, 0.9$ (x-axis) for patients in *group 2.* When patients in *group 1* have high drop-out propensity the median survival tends to be underestimated. Similarly, survival estimates tend to be overestimated when the drop-out probability is larger for patients with poor prognoses than for patients with good prognosis.

5) Potential misinterpretation of data summaries

Data summaries, such as posterior probabilities, p-values and confidence intervals, can be misinterpreted by patients and physicians. For example, a reported p-value equal to 0.2 may be interpreted as a 20% probability that the experimental treatment does not improve primary outcomes. Moreover, patients might not be familiar with uncertainty summaries (e.g. confidence intervals or probabilities). For some of them the propensity to enroll into a clinical trial could be highly reduced after the study release data summaries. The patient could be open to enroll into the trial in absence of early data summaries (impermeable trial), but could refuse enrollment if early summaries were released, unless these included strong evidence (say a posterior probability $> 90\%$) of clinically relevant treatment effects.

We consider a scenarios where a group of patients consider the experimental treatment in study j to be ineffective if $\pi_{j,\ell} < \pi_{IE}$ (in our simulations $\pi_{IE} = 0.7, 0.8$ or 0.9). These patients don't enroll into study j when $\pi_{j,\ell} < \pi_{IE}$. Figure S3 shows the average duration of

the permeable trial j_{prev} when the size p_0 of this group of patients varies between 0 and 0.5.

6) Heterogeneous enrollment rates across trials

In the permeable and impermeable model we assumed identical enrollment rates across trials when efficacy information is absent or when it coincides across trials (S3). In practice, there are significant differences in enrollment rates across trials for a variety of reasons independent of interim results. We evaluate departures from this assumption through study-specific parameters $(\gamma_j)_{j\geq 1}$ that modify the enrollment rates. The probability that a patient during the interval $[\ell, \ell+1)$ enrolls in trial j in the permeable environment becomes proportional to $\gamma_j \times g(\pi_{j,\ell})$. Before each simulation of a permeable environment, we generate trial specific parameters γ_j. The parameter γ_j then remains fixed during the trial j, and the overall enrollment rate λ_P, considering all open trials, remains constant.

We considered different degrees of variability of these baseline parameters γ_j across open trials. In our simulations we did not observe substantial changes in the operating characteristics of the permeable environment beyond the expected correlation between the parameters γ_j's and the corresponding trial-specific times to complete accrual.

7) Sensitivity to the parameters of the decision model

Simulations of clinical trials in a permeable environment require a model for the effects of early data summaries on the enrollment decisions. In our case, this model is defined by equation (S3). It may be possible to justify qualitative characteristics of the model, such as monotonicity of the probability that a patient selects a specific trial with respect to interpretable summary statistics. However, there is little knowledge about key parameters, for example θ_S in model (S3), which regulate how patients react to small variations of interim summary statistics, and the degree of homogeneity across patients of preferences and decisions. Additionally, it is likely that appropriate parameters for a specific disease, say a life threatening condition like Glioblastoma, might be unrealistic for a different pathology. Figure S2 illustrates variations in the operating characteristics when we consider different parameterizations θ in model (S3). Panel (A) illustrates sensitivity to the choice of θ_L on the power and probability of early termination of a study. Recall that in the simulation model trials can be stopped for insufficient accrual. As expected the probability of early termination of trials increases with θ_L. The panel illustrates also a monotone relation between θ_L and power (for j_{eff}) or type I error probability (for j_{prev}). Panel (B) of Figure S2 shows the average trial duration (for trials j_{eff} and j_{prev} as defined earlier) for different values of θ_S. As expected, the higher θ_S

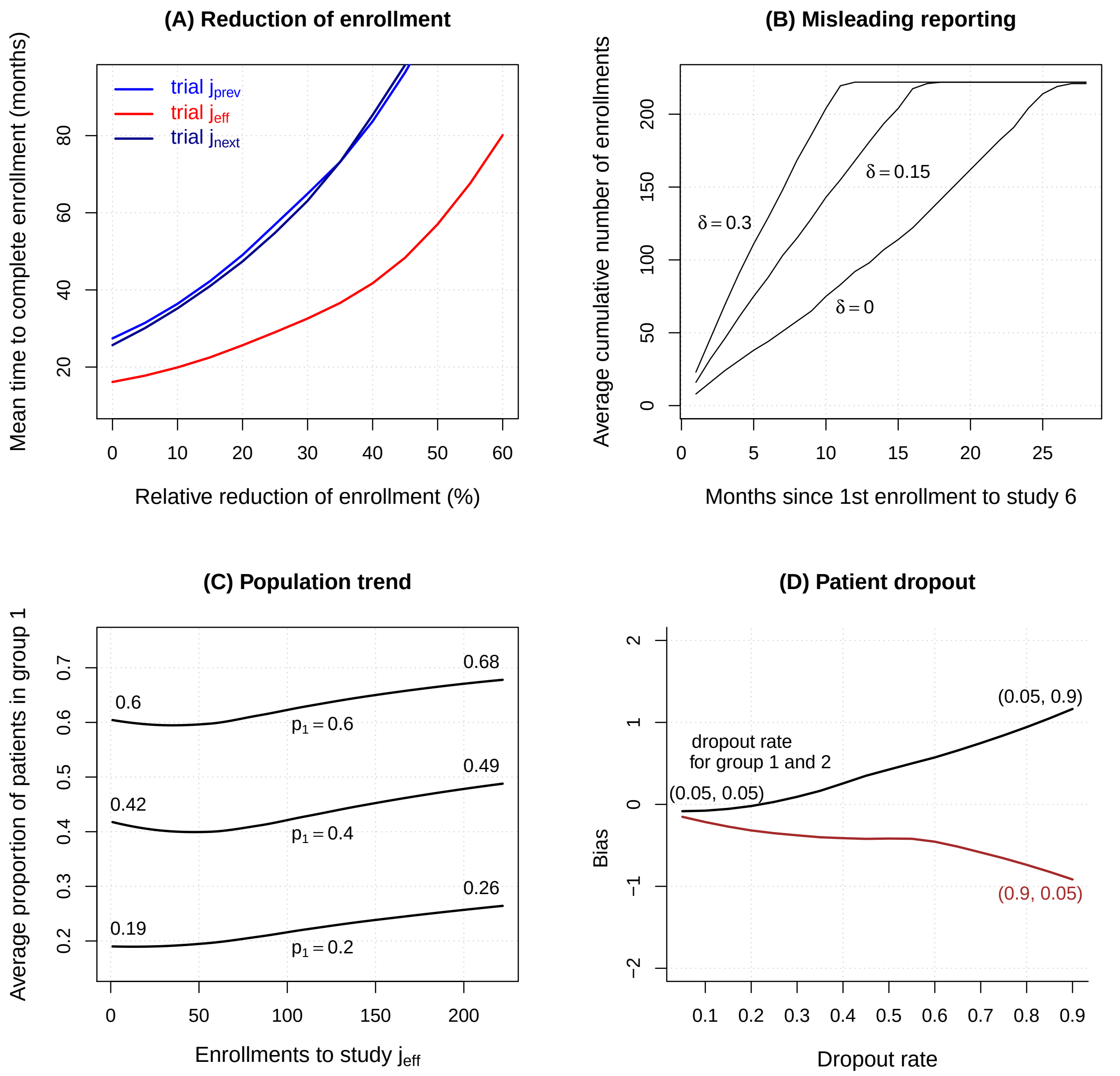

Figure S1: Sensitivity analysis in the permeable environment. Panel (A) shows the mean time to complete enrollment for a range of reduction parameters (x-axis) of the overall enrollment that are activated when $\max_{\text{active } j} \pi_{\ell,j} > 0.8$. Panel (B) shows the average cumulative number of enrollments for the study $j = 6$ (testing an ineffective treatment) during the accrual period with misreporting of summary statistics, $\pi_{\ell,j} \rightarrow \min(1, \pi_{\ell,j} + \delta_{bias})$, for $\delta_{bias} = 0, 0.15, 0.3$. Panel (C) shows the average proportion of enrolled patients in group 1 (y-axis) on study j_{eff} from the onset of the trial up to the i-th enrollment ($i = 1, \ldots, 244$) (x-axis). The proportion of patients in group 1 in the population equals $p_1 = 0.2, 0.4$ or 0.8. Panel (D) illustrates the average bias, i.e. the mean difference between estimated and true median survival time, for the experimental arm in trial j_{prev} when the drop-out probability of patients in group 1 (group 2) equals $p \in [0.05, 0.9]$ (x-axis).

the larger the difference becomes between the expected duration of competing trials testing effective and ineffective treatments. In this panel we assumed that arm 6 tests an ineffective treatment and compare it with j_{eff}.

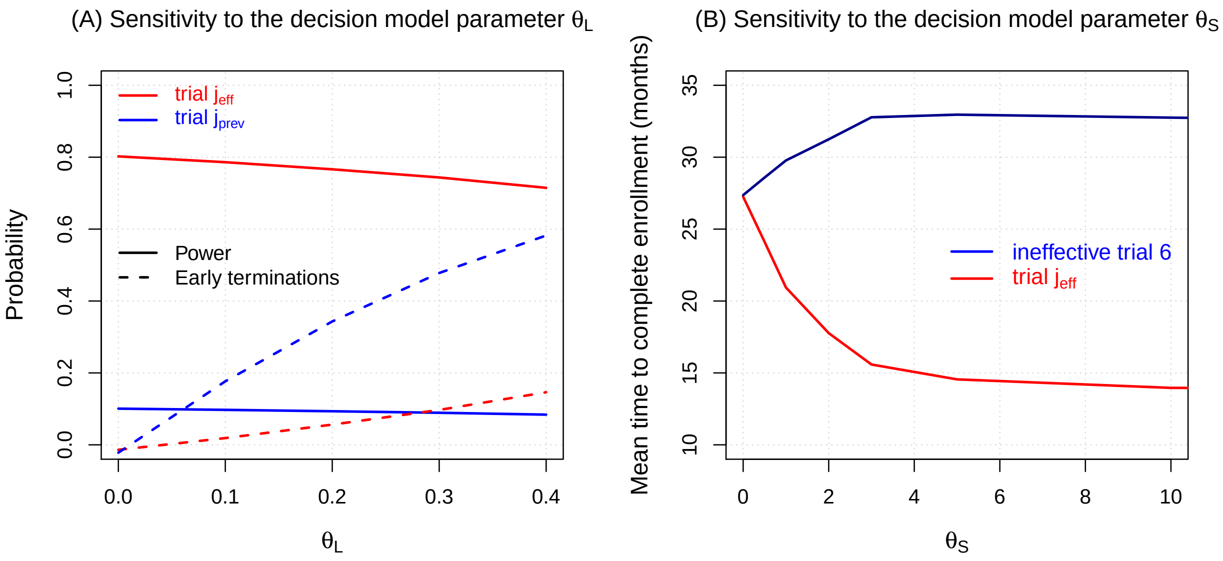

Figure S2: Sensitivity to patients decision-making model parameters. Panel (A) illustrates the sensitivity of power and the probability of early termination when we vary θ_L in model (S3). Panel (B) illustrates average trials' duration when we vary θ_S.

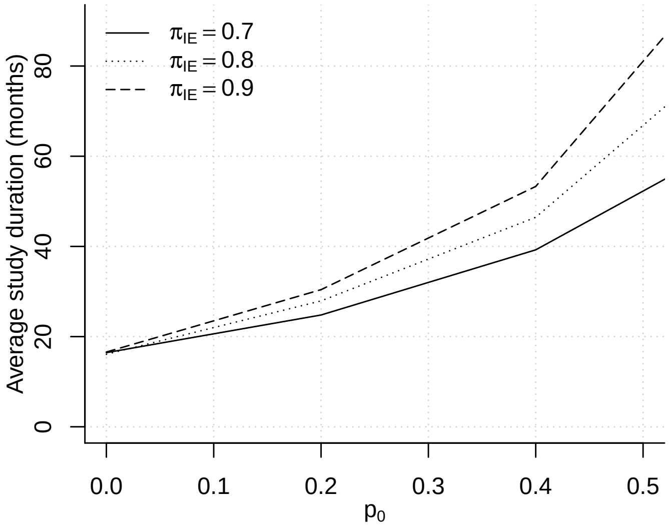

Figure S3: Sensitivity analysis: potential misinterpretation of early data summaries during permeable trials. The panel shows the average study duration (y-axis) of the permeable trial j_{prev} when $0 \leq p_0 \leq 0.5$.

S2 Permeability in Platform Trials

A platform trial is a multi-arm study that, by design, allows investigators to add and remove experimental arms as the trial progresses [1, 6, 7]. Arms can be added to the study when new treatments become available, and the number of arms may change in time. Experimental arms within platform designs can be compared during the study based on the available data. Platforms can potentially provide interpretable comparisons to clinicians or patients, which in turn can guide individual decisions.

We consider the following platform design. Patients are randomized to one of the active experimental arms or the control arm. In contrast to conventional platform trials each patient selects, before randomization, a list of arms and is then randomized to one of these arms with identical probabilities. Treatments on these lists are selected based on available information and personal preferences. The only requirements are the inclusion of the control arm and selection of at least one experimental arm.

In the permeable environment the available information influences patient decisions, with effects on the individual list of arms. In our model, during the period $[\ell, \ell+1)$ each patient i selects the experimental treatment j with probability

$$p_{j,\ell} = \begin{cases} \left(\dfrac{\pi_{j,\ell} - \theta_L}{\max\limits_{\text{active } s} \pi_{s,\ell} - \theta_L} \right)^{\theta_S} & \text{if } \pi_{j,\ell} > \theta_L, \\ 0 & \text{if } \pi_{j,\ell} < \theta_L, \end{cases} \tag{S2}$$

for each open experimental arm j. Here the arm j^* with the largest PTE probability is always included in the list, $p_{j^*,\ell} = 1$, whenever the posterior probability of a PTE is larger than θ_L. If all posterior probabilities $\pi_{j,\ell}$ of active experimental arms are smaller than θ_L, and therefore the patient's list does not include an experimental arm, the patient will not enroll in the platform study. As before, experimental arms that do not recruit patients for a pre-specified number of months are closed.

In the impermeable environment the individual list of treatments selected by patients does not relate to interim information. Patient i includes in the individual list an active arm j with probability $p_{IP} \in [0, 1]$, which is constant across arms. Only patients with lists that include one or more experimental arms in addition to the control are enrolled into the study. For the impermeable platform trial we include an early stopping rule for futility: to simplify comparisons with the permeable setting, if the probability $\pi_{j,\ell}$ for arm j falls below the threshold θ_L then arm j will be closed early.

To avoid selection bias, we apply the same strategy used in [6] and compare the outcome data of the experimental arm j only to those patients on the control arm that included arm j in their list of experimental treatments. These are the patients that could have been randomized to arm j with positive probability. We adopt this approach for permeable and in impermeable platforms. The method (restricted comparisons) combined with balanced randomization (i.e. identical randomization probabilities for the control arm and the experimental treatments that the patient selects), is robust with respect to potential variations of the populations [6] enrolled by the experimental and control arms during the platform study.

We evaluate the operating characteristics of permeable platform trials in a simulation study using the parameters of Table 1, discussed previously, which are based on a literature review of Glioblastoma trials. The platform starts with 5 experimental arms and up to 25 arms are added during a period of 10 years. Enrollment to an experimental arm j stopped if 230 patients, that selected arm j on their list, have been enrolled either to the experimental arm or the control arm.

For the permeable platform, we used $(\theta_L, \theta_S) = (0.05, 4)$ to define treatment selection probabilities (S2). With these parameter values patients on average include 55% of the active experimental arms in their randomization lists. For comparison purposes we assumed, similarly, that patients in the impermeable platform study include each available open arm within the randomization list with fixed probabilities equal to 0.55.

Table S3 summarizes selected operating characteristics of the platform trial designs. Permeable and impermeable platform trials have similar type I and II error rates, and a similar proportion of ineffective experimental treatments are stopped early for futility. Platform trials use a common control arm to evaluate experimental treatments in a single study. This, compared to conventional two-arm randomized clinical trial designs, reduces considerably the average time and the number of patients required for testing treatments [6]. Consequently, compared to two-arm trial environments, both platform environments — permeable and impermeable — reduce the average time to complete enrollment.

The simulation study for platform trials confirmed a relevant result which we obtained for permeable two-arm trials, indeed with permeable platform trials the time to complete enrollment for the first effective experimental treatment (indicated by j_{eff}) is shorter compared to ineffective treatments.

Study	Enrollment period Mean (SD)	Time to final results Mean (SD)	Arm specific Sample Size Mean (SD)	Power	P_{Stop}
		permeable platform trials			
ineffective drug j_{prev}	14.8 (10.8)	23.0 (08.9)	114.8 (16.4)	0.09	0.08
effective drug j_{eff}	09.6 (05.1)	23.4 (04.1)	110.8 (08.0)	0.81	<0.01
ineffective drug j_{next}	13.4 (09.5)	22.0 (07.7)	110.4 (16.6)	0.10	0.09
		impermeable platform trials			
ineffective drug j_{prev}	15.2 (04.8)	22.4 (04.3)	115.0 (07.9)	0.11	0.06
effective drug j_{eff}	14.9 (04.7)	25.5 (03.4)	112.6 (13.2)	0.80	<0.01
ineffective drug j_{next}	14.1 (04.5)	21.9 (04.1)	112.8 (12.7)	0.09	0.06

Table S3: Permeable and impermeable platform trials in Glioblastoma. Results are based on 5,000 simulations of a drug-development period of 10 years during which 30 experimental treatments are evaluated in a platform trial. We use $(\theta_L, \theta_S) = (0.05, 4)$ to define patients enrollment decisions (S2). Arm $j_{\text{eff}} \in \{6, \cdots, 27\}$ corresponds, in each simulation, to the first effective experimental treatment. Arm j_{prev} denotes the last experimental treatment that was added before arm j_{eff} was activated. Arm j_{next} corresponds to the first ineffective experimental treatment that opens enrollment after arm j_{eff}. Arms $j_{\text{prev}}, j_{\text{eff}}$ and j_{next} are random and vary across simulations.

References

[1] S. M. Berry, J. T. Connor, and R. J. Lewis. The platform trial: an efficient strategy for evaluating multiple treatments. JAMA, 313(16):1619–1620, 2015.

[2] D. Collett. Modelling survival data in medical research. CRC press, 2015.

[3] C. Jennison and B. W. Turnbull. Group sequential methods with applications to clinical trials. CRC Press, 1999.

[4] S. D. Robinson, J. A. O'Shaughnessy, C. L. Cowey, and K. Konduri. BRAF V600E-mutated lung adenocarcinoma with metastases to the brain responding to treatment with vemurafenib. Lung Cancer, 85:326–330, 2014.

[5] A. Vanderbeek, R. Rahman, F. Geoffrey, S. Ventz, T. Chen, R. Redd, G. Parmigiani, T. Cloughesy, P. Wen, L. Trippa, and B. Alexander. The clinical trials landscape for glioblastoma: is it adequate to develop new treatments? Neuro-Oncology, 2018.

[6] S. Ventz, B. M. Alexander, G. Parmigiani, R. D. Gelber, and L. Trippa. Designing clinical trials that accept new arms: An example in metastatic breast cancer. Journal of Clinical Oncology, 35(27):3160–3168, 2017. PMID: 28530853.

[7] S. Ventz, M. Cellamare, G. Parmigiani, and L. Trippa. Adding experimental arms to platform clinical trials: randomization procedures and interim analyses. Biostatistics, page kxx030, 2017.

Permeable environment

The probability $p_{j,\ell}$ that a patient selects study j, during month ℓ, is an increasing function of the the posterior probability of a positive treatment effect $\pi_{j,\ell} = \Pr(HR_j < 1|$ Data at month $\ell)$ where HR_j is the hazard-ratio between the experimental and control arm in study j. We compute the posterior probabilities $\pi_{j,\ell}$ for each open trial with a normal prior distribution for $\log(HR_j) \sim N(0, 1/2)$.

In our simulations, we assume that if $\pi_{j,\ell}$ is below a threshold $\theta_L \in [0,1]$, then patients do not enroll in trial j. Symmetrically, trials with $\pi_{j,\ell}$ above a threshold $\theta_U \in [\theta_L, 1]$ are selected with identical probabilities. The probability that a patient selects study j, during the period $[\ell, \ell+1)$, is $p_{j,\ell} \propto g_\theta(\pi_{j,\ell})$, where

$$g_\theta(\pi) = \begin{cases} 0 & \text{if } \pi < \theta_L, \\ \left(\frac{\pi-\theta_L}{\theta_U-\theta_L}\right)^{\theta_S} & \text{if } \pi \in [\theta_L, \theta_U], \\ 1 & \text{if } \pi > \theta_U, \end{cases} \tag{S3}$$

and $\theta_S \geq 0$. With θ_S large and θ_U close to one patients select the trial with the largest PTE.
